# Prediction of Ki-67 expression in bladder cancer based on CT radiomics nomogram

**DOI:** 10.3389/fonc.2024.1276526

**Published:** 2024-02-28

**Authors:** Shengxing Feng, Dongsheng Zhou, Yueming Li, Runqiang Yuan, Jie Kong, Feng Jiang, Weitian Chen, Lijie Zhang, Mancheng Gong

**Affiliations:** ^1^ Reproductive Center, Maoming People’s Hospital, Maoming, China; ^2^ The First Clinical School of Medicine, Guangdong Medical University, Zhanjiang, China; ^3^ Department of Urology, The People’s Hospital of Zhongshan, Zhongshan, China

**Keywords:** bladder cancer, Ki-67, radiomics, nomogram, computed tomography (CT)

## Abstract

**Objectives:**

This study aimed to create and validate a radiomics nomogram for non-invasive preoperative Ki-67 expression level prediction in patients with bladder cancer (BCa) using contrast-enhanced CT radiomics features.

**Methods:**

A retrospective analysis of 135 patients was conducted, 79 of whom had high levels of Ki-67 expression and 56 of whom had low levels. For the dimensionality reduction analysis, the best features were chosen using the least absolute shrinkage selection operator and one-way analysis of variance. Then, a radiomics nomogram was created using multiple logistic regression analysis based on radiomics features and clinical independent risk factors. The performance of the model was assessed using the Akaike information criterion (AIC) value, the area under the curve (AUC) value, accuracy, sensitivity, and specificity. The clinical usefulness of the model was assessed using decision curve analysis (DCA).

**Results:**

Finally, to establish a radiomics nomogram, the best 5 features were chosen and integrated with the independent clinical risk factors (T stage) and Rad-score. This radiomics nomogram demonstrated significant correction and discriminating performance in both the training and validation sets, with an AUC of 0.836 and 0.887, respectively. This radiomics nomogram had the lowest AIC value (AIC = 103.16), which was considered to be the best model. When compared to clinical factor model and radiomics signature, DCA demonstrated the more value of the radiomics nomogram.

**Conclusion:**

Enhanced CT-based radiomics nomogram can better predict Ki-67 expression in BCa patients and can be used for prognosis assessment and clinical decision making.

## 1 Introduction

One of the diseases in the top 10 most prevalent worldwide is bladder cancer (BCa). Approximately 20% of newly diagnosed bladder cancers remain to be muscle-invasive bladder cancer (MIBC), and approximately 50% of patients with MIBC who undergo radical cystectomy (RC) with pelvic lymphadenectomy have distant metastases (1, 2). Despite curative local therapy, 50%–60% of patients with MIBC will continue to experience metastatic progression (3). In addition, BCa has the characteristics of easy recurrence, rapid progression, and easy metastasis (4), which brings great difficulties to our precision and individualized treatment.

All cells in the cycle express the proliferation-associated nuclear antigen Ki-67, with the exception of dormant cells in the g0 phase (5). Ki-67 protein is widely used as a proliferation marker reflecting the number of cells in a tumor (6). According to prior research, increased expression of Ki67 has been linked to a poor outcome for bladder tumors (7), tumor grade, tumor T stage (8), recurrence (9), and lymph node metastasis (10). At the same time, studies have shown that PD-L1 expression is closely related to Ki-67 positivity (11). Patients with non-muscle-invasive bladder cancer receiving intravesical immunotherapy with BCG have a higher risk of progression-free survival (PFS) if their Ki-67 levels are high (12). In conclusion, Ki-67 expression may be a useful indicator of immunotherapy effectiveness and response in BCa, in addition to serving as a reference index for clinical metastatic disease and patient prognosis. In BCa, Ki-67 expression is generally detected by immunohistochemistry in selected cystoscopic biopsies or postoperative specimens from radical surgery. However, because BCa specimens have a variety of tumor types and immunohistochemical detection uses a small sample size, the final Ki-67 expression cannot represent the whole BCa tissue, which limits the accuracy and application of this analysis. In addition, immunohistochemical staining takes 3–5 days, so it is not possible to evaluate Ki-67 index in real time. Therefore, it is crucial to look for a quick, reliable, and comprehensive method of predicting Ki-67 expression in BCa patients.

Radiomics is an image feature extraction technique based on automated algorithms, which can quickly and accurately obtain image information of different regions in a non-invasive manner, and can effectively reflect the heterogeneity of lesions (13, 14). Radiomics technology provides a new idea for quantitative evaluation of tumor heterogeneity and shows great advantages in clinical practice. The pathophysiology of bladder tumors can be evaluated using radiomics with a high degree of accuracy, according to prior studies, including the differentiation of benign and malignant (15), tumor invasion (16, 17) and tumor recurrence (18). In predicting Ki-67 expression in breast cancer (19) and liver cancer (20), radiomics has some advantages. To the best of the knowledge, it is the first the CT-based research that examines to whether radiomics can be employed as a tool to determine the status of Ki-67 expression in BCa. In order to support clinical decision making, we developed and validated a radiomics nomogram to forecast Ki-67 expression in BCa patients.

## 2 Materials and methods

### 2.1 Patients

**Figure 1:**
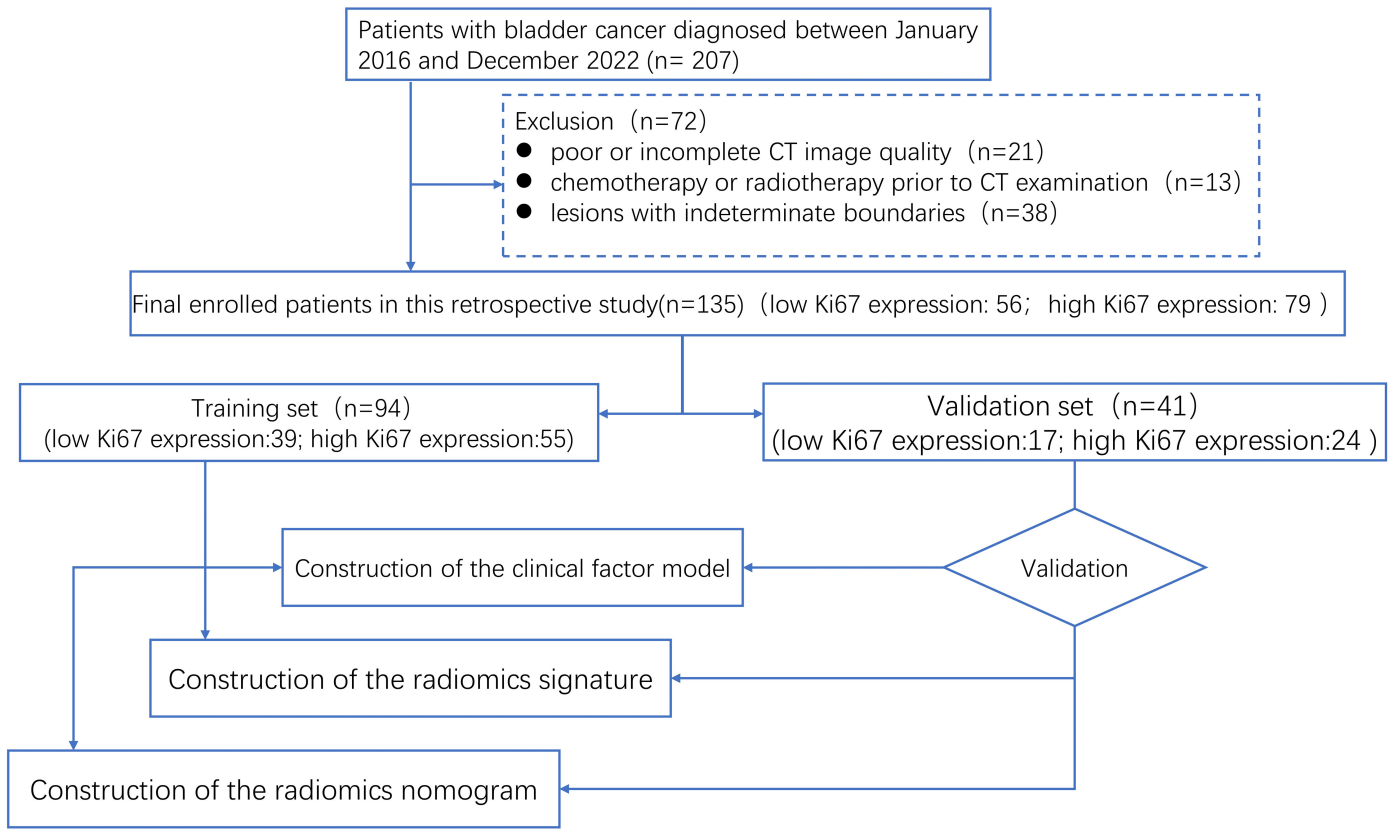
The study flow chart of the study.

Our workflow is shown in **Figure 1**. In this retrospective cohort research, between January 2016 and December 2022, patients with BCa who had been diagnosed by pathology at our hospital were gathered. The following criteria were used for inclusion: (1) BCa sufferers who have transurethral resection or radical cystectomy; (2) BCa diagnosed by histopathology and immunohistochemistry and BCa stage shown to correlate with Ki-67 expression; (3) preoperative enhanced CT examination; and (4) complete Ki-67 expression and prognostic data. Among the exclusion criteria were (1) poor or incomplete CT image quality, (2) chemotherapy or radiotherapy prior to CT examination, and (3) lesions with indeterminate boundaries. In the end, all 135 patients were included in the research. A total of 135 patients were randomly assigned to training and validation sets in a 7:3 ratio (21).

### 2.2 Image acquisition (instruments and methods)

A 256-slice spiral CT-enhanced scan (Brilliance, Philips Healthcare) was performed on all patients. The following device specifications were used: collimation, 64×0.6 mm; tube voltage, 120 kV; automatic modulation of activated tube current; pitch, 0.9; image matrix, 512×512; and slice thickness/slice spacing, 3 mm/3 mm. From the transverse septum to the pelvic floor, patients underwent scans, and iopamidol 100 mL or ioversol 80 mL was intravenously injected, followed by saline 50 mL at a speed of 3 mL/s. At 30, 60, and 300 s following threshold, images of the corticomedullary phase, nephrogram phase, and excretory phase were obtained.

### 2.3 Evaluation of Ki-67 expression

An expert pathologist evaluated each specimen using immunohistochemistry. The kit’s instructions were followed for doing immunohistochemistry with mouse anti-human Ki-67 monoclonal antibody from Shanghai Gene Technology Co., Ltd. Cells with brown nuclei are considered positive. Positive cells were chosen from the five areas with the highest the positive density, and the portion of positive labeled cells was calculated using a high-power microscope (×400). The Ki-67 index used in this study was the average of the five locations with the highest proportion of Ki-67 labeled cells. According to previous research (7, 22–24), BCa patients were separated into two groups based on their Ki-67 expression levels: high expression (>15% cell staining) and low expression (≤15% cell staining).

### 2.4 CT image delineation and feature extraction

**Figure 2:**
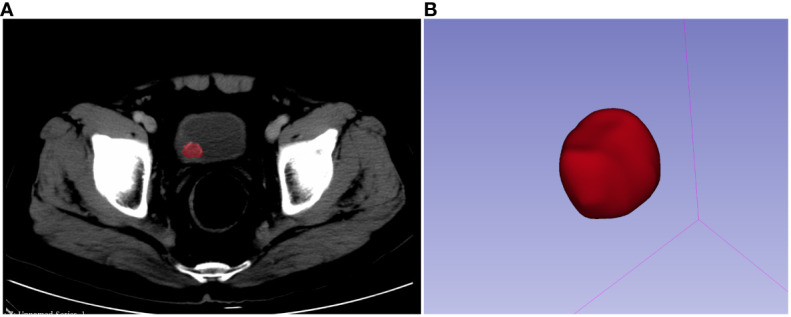
Manual segmentation of the mass **(A)** and three-dimensional volumetric reconstruction **(B)**.

Before contouring, each and every CT image was resampled to a voxel size of 1×1×1 mm, discretized to grayscale preprocessing, and given a bandwidth of 25. A three-dimensional (3-D) region of interest (ROI) was drawn on the image not more than 1 mm along the lesion margin using 3D slicer software (version: 4.10.2.). A segmentation example is shown in **Figure 2**. For each patient, 107 picture characteristics in total, including 14 form features, 18 first-order features, and 75 texture features were retrieved. In the end, 1,223 radiomics features were obtained by LoG (σ: 0.5, 1.0, 1.5, and 2.0) and wavelet. About the set of radiology function for more information, please visit https://pyradiomics.readthedocs.io/en/latest/#. Data were then Z-score normalized before further analysis.

### 2.5 Observation of feature consistency

The repeatability of feature extraction was assessed using the inter- and intra-class correlation coefficients (ICCs). Radiologist A and radiologist B selected 20 lesions at random from the training set and delineated them to evaluate the reliability of interobserver features. Radiologist A delineated 20 lesions again 3 weeks later, and the first time that the features were extracted were compared to determine the intra-observer reliability. ICC > 0.75 was considered to have good consistency and stability of the selected features. Radiologist A will delineate the remaining lesions and extract features.

### 2.6 Feature selection and radiomics signature construction

First, to choose features with significant differences between high and low expression of the Ki-67 index, features with ICC > 0.75 were assessed by one-way analysis of variance (ANOVA). The least absolute shrinkage selection operator (LASSO) regression method was used to further select the key radiomics features with nonzero coefficients, and a 10-fold cross-validation was performed to determine the optimal modulation weights. The final radiomics features were chosen, and their corresponding coefficients were multiplied to create the radiomics score (Rad-score) for each patient, and finally, the radiomics signature was constructed.

### 2.7 Construction and model validation of radiomics nomogram

Using CT imaging features and clinical features from the training set as clinical variables, univariate and multivariate logistic regression analyses were done to identify independent predictors of Ki-67 expression level in BCa lesions, and a clinical factor model was developed. Then, by integrating Rad-score and clinical independent predictors, a radiomics nomogram was created. The validation set validated each model’s performance. We evaluated the predictive performance of the clinical factor model, radiomics signature, and radiomics nomogram in the training and validation sets based on sensitivity, specificity, accuracy, and area under curve (AUC) values. To compare the variations between them, the Delong test was employed. The Hosmer–Lemeshow test and calibration curve were used to confirm the nomogram model’s consistency. The Akaike information criterion (AIC) was employed to gauge how well the model fit the data. In addition, in order to assess the clinical utility of three models in predicting Ki-67 expression, decision curve analysis (DCA) was performed.

### 2.8 Statistical analysis

R software 4.2.0 was used to conduct statistical analysis on the data. Fisher’s exact test or chi-square test were used to compare qualitative data, while the t-test or Mann–Whitney U test were used to compare quantitative data, as applicable. The “rms” package was used to construct nomograms and calibration curves. The Hosmer–Lemeshow test is carried out using the “generalhoslem” package. We used “glmnet” for LASSO regression model analysis. We used the “pROC” software to plot the analytical ROC curve. The Delong test was employed to calculate how much the three models’ AUC values varied from one another. We used the software program “DCA.r” to perform DCA. Statistical significance was set at *p* < 0.05.

## 3 Results

### 3.1 Clinicopathological features

After screening, finally, 135 patients were enrolled in this trial; 108 of them were men, and 27 were women. The average age was 66.16 ± 12.67 years, and the average tumor size was 23.53 ± 14.96 mm. **Table 1** displays specific clinical information for the groups with low and high Ki-67 expression. **Table 2** displays the clinical features information from the training set and validation set. A clinical factor model was created after additional univariate and multivariate logistic regression analyses revealed that only clinical T stage was an independent risk factor for elevated Ki-67 expression (**Table 3**).

**Table 1:** Clinical data of bladder cancer patients in low Ki-67 group and high Ki-67 group.

Clinical factor	Low Ki-67 (n=56)	High Ki-67 (n=79)	t/X2	*p*
Age (year)	62.12 ± 14.77	69.03 ± 10.9	−3.23	0.002
Gender (male/female)	44/12	64/15	0.017	0.896
Tumor size (mm)	17.86 ± 12.58	27.55 ± 15.28	−3.90	<0.001
Height (cm)	163.65 ± 8.58	162.37 ± 6.89	0.959	0.340
Weight (kg)	62.50 ± 11.15	62.05 ± 11.79	0.223	0.822
BMI (kg/m2)	23.23 ± 3.05	23.45 ± 3.57	−0.378	0.706
Clinical T stage (T2/≥T2)	53/3	52/27	14.125	<0.001
PLR	140.56 ± 48.28	141.63 ± 56.42	−0.115	0.908
NLR	2.44 ± 1.01	2.53 ± 1.10	−0.481	0631

**Table 2:** The clinical data between low and high Ki-67 group in the training and validation cohorts.

Clinical factors	Training set (n = 94)			Validation set (n = 41)		
	Low Ki-67	High Ki-67	*P*	Low Ki-67	High Ki-67	*p*
Age (year)	63.82 ± 15.26	69.55 ± 9.91	0.030	58.24 ± 13.20	67.83 ± 10.61	0.014
Gender (male/female)	31/8	47/8	0.631	13/4	17/7	0.736
Tumor size (mm)	18.95 ± 14.03	26.68 ± 13.78	0.009	15.35 ± 8.20	29.54 ± 18.44	0.005
Height(cm)	163.72 ± 8.92	162.83 ± 6.54	0.578	163.50 ± 8.00	161.33 ± 7.69	0.387
Weight (kg)	63.67 ± 11.70	62.43 ± 11.78	0.615	59.82 ± 9.58	61.17 ± 12.01	0.703
BMI (kg/m2)	23.60 ± 2.99	23.44 ± 3.52	0.809	22.36 ± 3.10	23.48 ± 3.78	0.322
Clinical T stage (<T2/≥T2)	36/3	34/21	0.002	17/0	18/6	0.033
PLR	138.76 ± 47.69	138.78 ± 53.94	0.999	144.68 ± 50.86	148.17 ± 62.46	0.850
NLR	2.41 ± 1.03	2.50 ± 1.08	0.662	2.53 ± 0.97	2.60 ± 1.18	0.838

PLR, platelet-lymphocyte rate; NLR, neutrophil-lymphocyte ratio.

**Table 3:** Univariate and multivariate logistic regression analysis of the clinical factors.

Characteristics	Univariate analysis			Multivariate analysis		
	OR	95% CI	*P*	OR	95% CI	*P*
Age	1.038	1.004–1.077	0.036	1.045	1.003–1.056	0.052
Tumor size	1.048	1.012–1.091	0.014	0.977	0.931–1.026	0.342
Clinical T stage (<T2/≥T2)	7.412	2.294–33.417	0.002	5.909	1.554–30.551	0.016
Rad-score	2.718	1.676–5.014	<0.001	2.914	1.590–6.312	0.002
Gender (male/female)	0.660	0.220–1.969	0.450			
Height	0.984	0.931–1.040	0.573			
Weight	0.991	0.956–1.027	0.611			
BMI	0.984	0.867–1.117	0.806			
PLR	1.000	0.992–1.008	0.999			
NLR	1.094	0.739–1.663	0659			

CI, confidence interval; OR, odds ratio; PLR, platelet–lymphocyte rate; NLR, neutrophil–lymphocyte ratio.

### 3.2 Radiomics feature extraction and analysis

**Figure 3:**
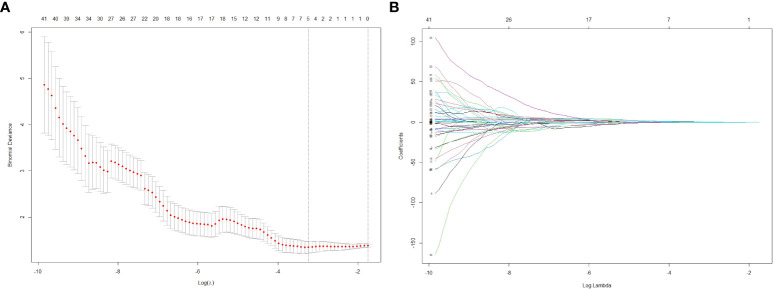
The LASSO regression model was used to select 5 radiomics features with significant discrimination significance. The optimal value of LASSO tuning parameter (λ) is represented by the vertical dashed line. The best adjustment of the regularization parameter λ to 0.039 under the minimum criterion was found by ten-fold cross validation **(A)**. LASSO coefficient profiles of the radiomics features **(B)**.

From each patient’s CT scans, 1,223 radiomics features were collected, and 20 patients were randomly selected for ICC analyses, and for further investigation, characteristics with high reproducibility and stability (ICC > 0.75) were chosen. Features dimension reduction was performed by one-way ANOVA and LASSO review in turn. Finally, five radiomics features with important discrimination significance were selected (**Table 4**), and the best adjustment regularization parameter (λ = 0.039) under the minimum standard was found by 10-fold cross-validation (**Figure 3**) to construct the radiomics signature. This formula was used to determine the Rad-score:

**Table 4:** List of the selected features with non-zero coefficients.

Image Type	Feature Class	Feature Name
original	shape	Minor Axis Length
log-sigma-0-5-mm-3D	ngtdm	Busyness
wavelet-LHH	gldm	Small Dependence Emphasis
wavelet-LHH	glszm	Large Area Low Gray Level Emphasis
wavelet-HHL	glszm	Gray Level Non Uniformity

Rad-score=0.838 + 0.379*original.shape.Minor.Axis.Length+0.044*log-sigma-0-5-mm-3D.ngtdm.Busyness-1.005*wavelet-LHH.gldm.Small Dependence Emphasis-0.139*wavelet-LHH.glszm.Large Area Low Gray Level Emphasis+0.023*wavelet-HHL.glszm.Gray Level Non Uniformity.

As shown in **Table 5**, the AUC of the radiomics signature in the training and validation sets were 0.821 (95% CI, 0.734–0.908) and 0.877 (95% CI, 0.761, 0.994), respectively.

**Table 5:** Diagnostic performance of the clinical factor model, the radiomics signature, and the radiomics nomogram.

Model	Training set (n=94)				Validation set (n=41)			
	AUC (95% CI)	Sensitivity	Specificity	Accuracy	AUC (95% CI)	Sensitivity	Specificity	Accuracy
Clinical factor model	0.652(0.575, 0.730)	0.382	0.923	0.606	0.625(0.537, 0.713)	0.250	1.000	0.561
Radiomics signature	0.821(0.734, 0.908)	0.873	0.641	0.777	0.877(0.761, 0.994)	0.958	0.647	0.829
Radiomics nomogram	0.836(0.751, 0.921)	0.855	0.667	0.777	0.887(0.780, 0.995)	0.833	0.824	0.829

CI, confidence interval.

### 3.3 Construction of a nomogram and evaluation of the efficacy of several models

**Figure 4:**
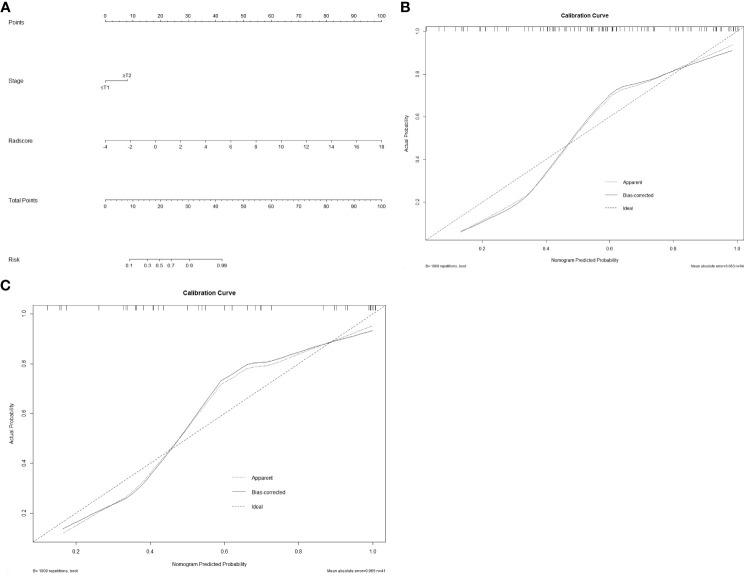
The radiomics nomogram and calibration curves. Based on the identified statistically significant clinical factor (clinical T stage) and Rad-score, a radiomics nomogram was established by multivariate logistic regression analysis **(A)**. Calibration curves of the nomogram in the training **(B)** and validation **(C)** sets. The 45° straight line represents the ideal forecast. The closer the two curves are, the higher the accuracy will be.

A radiomics nomogram was created using multivariate logistic regression analysis based on the clinical factor that were determined to be statistically significant (clinical T stage) and the Rad-score (**Figure 4A**). The calibration curve and Hosmer–Lemeshow test demonstrated strong calibration in both the training set (*p* = 0.098) and the validation set (*p* = 0.272) (**Figures 4B, C**), indicating good agreement between predictions and observations in both sets.

**Figure 5:**
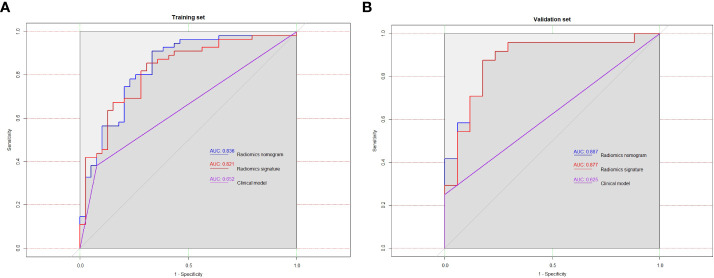
In training set **(A)** and validation set **(B)**, the ROC curves of the three models were plotted, respectively.


**Table 5** summarizes the prediction performance of the clinical factor model, radiomics signature, and radiomics nomogram on Ki-67 expression level in the training set (AUC = 0.652, 0.821, and 0.836) and validation set (AUC = 0.625, 0.877, and 0.887). **Figures 5A, B** display the ROC curves for the three models in the training and validation sets. The radiomics nomogram considerably outperformed the clinical factor model in both the training and validation sets (*p* < 0.001) with a higher AUC. However, the radiomics nomogram and radiomics signature’s AUC values did not differ significantly in the training and validation sets (*p* = 0.6123, 0.3047). When comparing radiomics signature with clinical factor model, radiomics signature had a higher AUC in the training and validation sets than the clinical factor model (*p* = 0.0038, 0.0002). The following formula was used to determine the Nomo-score:

$$\text{Nomo}-\text{score}=1.6097^{*}\text{ clinical T stage }+0.9215^{*}\text{ rad}-\text{score }-0.2862$$

**Figure 6:**
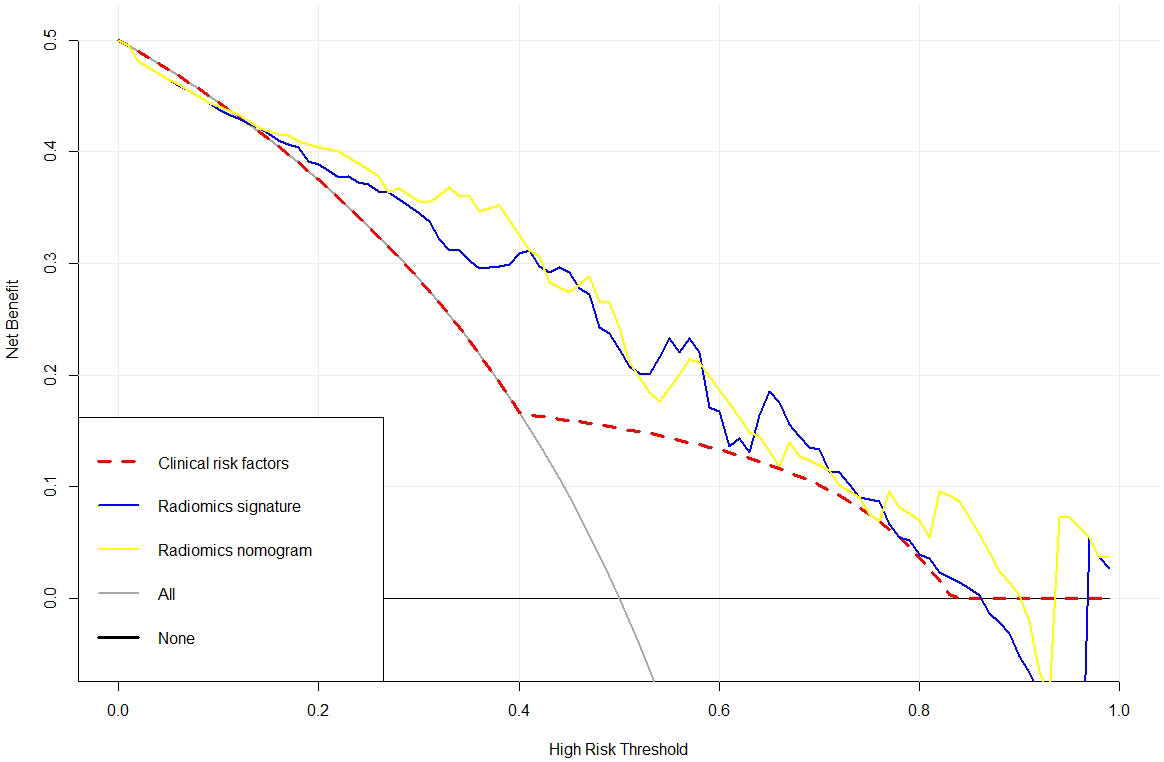
An analysis of decision curves for three models.

Meanwhile, in comparison to the clinical factors model (AIC = 119.07) and the radiomics signature (AIC = 107.51), the radiomics nomogram (AIC = 103.16), which had the smallest AIC value, was deemed to be the most effective model. The three models in the training set’s clinical utility were evaluated using DCA (**Figure 6**). Compared to the clinical factor model or the radiomics signature, the radiomics nomogram was better at predicting the levels of Ki-67 expression and offered higher net benefit over a broader spectrum of risk thresholds.

## 4 Discussion

In our research project, a radiomics nomogram based on enhanced CT was created and affirmed for non-invasive prediction of Ki-67 protein level in BCa. It includes clinical factor (clinical T stage) and Rad-score. Comparing the radiomics nomogram to the clinical factor model or the radiomics signature, the radiomics nomogram enhanced the prediction of Ki-67 protein level. In both the training and validation sets, the established radiomics nomogram demonstrated good discriminatory practices (AUC = 0.836 and 0.887). To the best of our knowledge, this is the first study to predict Ki-67 expression status in bladder cancer based on CT radiomics nomogram. The results showed that the radiomics nomogram showed good accuracy and practicality in predicting the expression level of Ki-67 protein, which may have potential application value for clinical decision making of BCa.

Ki-67 protein is a nuclear protein with proliferative activity found in tumors at many stages of cell, with the exception of quiescent cells in the g0 phase (25). Previous research has revealed that the high expression of Ki-67 may be associated with the malignant degree, invasive behavior, cell proliferation activity, immunotherapy, and poor prognosis of BCa. In a study by Wang et al., 313 patients with non-muscle invasive BCa were collected and analyzed, and the Ki-67 marker index was correlated with the pathological grade, clinical stage, tumor size, and number of BCa (6). In a study by Rubino et al., in which 130 patients with MIBC were analyzed based on the construction of tissue microarrays, regression modeling findings indicated that elevated expression of Ki-67 was closely connected with positive PD-L1 expression, from which it can be concluded that elevated expression of Ki-67 may be connected with immunotherapy for BCa. In addition, the regression model results showed that elevated expression of Ki-67 was strongly connected with poorer overall survival (11). By collecting 213 patients with papillary BCa, March-Villalba et al. revealed that the Ki-67 index improves the prediction of recurrence and PFS in papillary BCa (23). Similarly, Liu et al. showed that elevated expression of Ki-67 predicted bad prognosis in BCa patients by collecting 84 patients who underwent surgery (26). Culpan et al. included 101 patients with primary BCa, and the results suggested that, in pT1 BCa patients, the biomarker Ki-67 significantly predicted recurrence-free, progression-free, and cancer-specific survival (27).

CT is currently a common and important method for diagnosing BCa. In recent years, radiomics, as an emerging and popular medical clinical aid and diagnostic technique, can obtain radiomics features from a large number of medical imaging images to identify tumor heterogeneity (28) and can obtain quantitative information on tumor lesion size, shape, texture, and heterogeneity beyond visual assessment to filter the most valuable radiomics features of interest and construct predictive models (29). Previously, the viability and efficiency of radiomics in preoperative prediction of Ki-67 expression in malignancies have been shown in a variety of research. By collecting 339 patients with gastrointestinal stromal tumor (GIST) from four centers, Zhang et al. concluded that the radiomics features of CT were significantly connected with the levels of Ki-67 in GIST, and the AUC of external verification was as high as 0.784 (30). The levels of Ki-67 were predicted using the CT radiomics nomogram by Zheng et al. after collecting 217 patients with head and neck squamous cell carcinoma. The model demonstrated good prediction effect with AUC of 0.919 (31). Bao et al., with 206 lung adenocarcinoma patients, built a radiomics nomogram to forecast Ki-67 proliferation index; AUC is 0.91 (32).

Nevertheless, to the authors’ understanding, this research is the only study so far to investigate whether CT radiomics can be applied as an instrument to estimate the Ki-67 expression state in BCa patients. As in research, we developed and validated a radiomics nomogram stem from enhanced CT to forecast Ki-67 expression index on BCa patients. In this study, we sketched a 3D ROI, which is more comprehensive and provides a better understanding of tumor heterogeneity compared to 2D (33). In addition, we extract features by wavelet, which are not available from conventional texture analysis. We acquired a large number of the most valuable radiomics features from CT images and added clinical characteristics predictive factors for risk to build radiomics nomogram. Our study showed that the radiomics nomogram could well predict the expression of Ki-67 in BCa patients.

Additionally, this research has several drawbacks. First off, this was a retrospective study with some selection bias in enrolled patients. Furthermore, this research’s number of participants was somewhat limited, and there was no external validation. As a result, additional validation efforts involving larger samples, external validation, and multicenter investigations are required. Third, this study did not explore the expression of Ki-67 in various clinical subtypes; future research may examine this issue more.

## 5 Conclusion

To sum up, this radiomics nomogram incorporating pathological with radiomics features showed good forecasting accuracy and clinical application for forecasting Ki-67 expression levels in BCa patients. As a non-invasive method, it can provide important support for personalized treatment and prognosis evaluation of patients with BCa.

## Data availability statement

The raw data supporting the conclusions of this article will be made available by the authors, without undue reservation.

## Ethics statement

The studies involving humans were approved by The Ethics Committee of Zhongshan City People’s Hospital (approval code: 2023-012). The studies were conducted in accordance with the local legislation and institutional requirements. The requirement for informed consent was waived due to the retrospective nature of the study.

## Author contributions

MG: Supervision, Writing – review & editing. SF: Conceptualization, Data curation, Methodology, Software, Writing – original draft. DZ: Conceptualization, Investigation, Methodology, Software, Visualization, Writing – original draft. YL: Writing – review & editing. RY: Writing – review & editing. JK: Investigation, Visualization, Writing – original draft. FJ: Data curation, Writing – original draft. WC: Software, Validation, Writing – original draft. LZ: Software, Validation, Writing – original draft.
